# Simulation of avascular tumor growth by agent-based game model involving phenotype-phenotype interactions

**DOI:** 10.1038/srep17992

**Published:** 2015-12-09

**Authors:** Yong Chen, Hengtong Wang, Jiangang Zhang, Ke Chen, Yumin Li

**Affiliations:** 1 Key Laboratory of Digestive System Tumors of Gansu Province, Lanzhou University, Lanzhou 730000, China; 2 Center of Soft Matter Physics and its Applications, Beihang University, Beijing 100191, China; 3 School of Physics and Information Technology, Shaanxi Normal University, Xi’an 710119, China; 4 Institute of Pathology, Lanzhou University, Lanzhou 730000, China; 5 Key Laboratory of Genome Sciences and Information, Beijing Institute of Genomics, Chinese Academy of Sciences, Beijing 100101, China

## Abstract

All tumors, both benign and metastatic, undergo an avascular growth stage with nutrients supplied by the surrounding tissue. This avascular growth process is much easier to carry out in more qualitative and quantitative experiments starting from tumor spheroids *in vitro* with reliable reproducibility. Essentially, this tumor progression would be described as a sequence of phenotypes. Using agent-based simulation in a two-dimensional spatial lattice, we constructed a composite growth model in which the phenotypic behavior of tumor cells depends on not only the local nutrient concentration and cell count but also the game among cells. Our simulation results demonstrated that in silico tumors are qualitatively similar to those observed in tumor spheroid experiments. We also found that the payoffs in the game between two living cell phenotypes can influence the growth velocity and surface roughness of tumors at the same time. Finally, this current model is flexible and can be easily extended to discuss other situations, such as environmental heterogeneity and mutation.

Tumors are a class of cell populations growing out of control because of an abnormality of the cell cycle (evasion of cell apoptosis) induced by the environment or genetic mutation^1^,^2^. Tumors can be classified into two types, benign tumors or malignant tumors. Sometimes, the benign tumors become cancerous resulting from mutation. Today, cancer still has a very high death rate despite remarkable advances in understanding its genomic changes^3^.

In the past decade, simulation has served as a powerful tool to study the cancer behaviors across different biological scales in space and time has increasingly attracted the interest of researchers^4^,^5^. Correspondingly, modeling techniques are roughly classified as continuum, discrete, and hybrid. Continuum models are used to study large-scale systems by treating the tumor tissue as a continuous medium^6^,^7^,^8^,^9^,^10^,^11^,^12^. Tumor behaviors are described by several variables and partial/ordinary differential equations (PDEs/ODEs) with the principle of mass conservation. These models catch the global properties of the tumor but lose the microscopic individual cell dynamics and microenvironment. However, discrete modeling can track individual cell dynamics using an agent-based model with a set rules in a lattice or lattice-free space^13^,^14^,^15^. These models are a good choice for reflecting cell phenotypes and microenvironment but are limited by the computational demand increasing rapidly with the cell count. The hybrid models integrate both continuum and discrete descriptions and have potential across multiple scales from the molecule and cell to the tissue^16^,^17^,^18^. It is very worth noting that a three-dimensional spatial model has been recently successfully constructed to study the tumor growth and the intratumour heterogeneity^19^.

Tumor cells have heterogeneous properties at the genotypic and phenotypic levels. In addition, the tumor cells compete with other tumor cells and with healthy cells and the physical microenviornment for space and resources^20^. Giese *et al*. found that migration and proliferation cannot simultaneously occur in one glioma cell^21^. The cell phenotype may switch to another because of mutation or metabolic stress^22^. Thus, this cell adaptation is very suitable for using use evolutionary game theory (EGT) to model the tumor dynamics as well as ecology^23^,^24^,^25^.

EGT has been used to address many aspects of cancer biology^26^,^27^,^28^,^29^,^30^,^31^. Gatenby *et al*. demonstrated that carcinogenesis requires cellular proliferation to successfully adapt varying environmental constraints based on population biology and game theory^32^,^33^,^34^. Some researchers consider tumor metabolism in the evolutionary game sense^35^,^36^. Tomlinson *et al*. constituted a simple angiogenic game for the cell strategies of producing growth factor or not^37^. The Warburg effect also can be explained by the Prisoner′s Dilemma game between aerobic and glycolytic cells with measurements of metabolic efficiency and competitive ability^38^,^39^. Basanta *et al*. studied the edge effects with the game between autonomous growth and motility and found that spatial structure can enhance invasive ability^40^. More importantly, evolutionary dynamics have been applied to clinical therapy, radiation and drug treatments^32^,^41^,^42^,^43^,^44^.

Tumor always grow from a small number of malignant proliferative cells and goes through an initial avascular stage of growth. To study avascular tumor growth and malignant development (for example, invasion, angiogenesis, etc.), it is very valuable to better understand tumor progression and metastasis. Although genetic mutations and the microenvironment are much more complicated, the tumor growth process would be reliably illustrated by several cell phenotypes^34^,^40^,^45^,^46^.

In this study, we focus on the effect of phenotype-phenotype competition on the properties of avascular tumor growth. Inspired by the game between different cell phenotypes and the reaction-diffusion model of tumor growth^45^,^47^, we propose a composite agent-based model involving the game among phenotypes and the effect of local nutrient concentration. Considering the similar payoff parameters as suggested by Mansury *et al*.^45^, we present extensive simulations to analyze the structural and dynamic properties in the growth process of avascular tumors.

## Results

### Model Description

**Figure 1:**
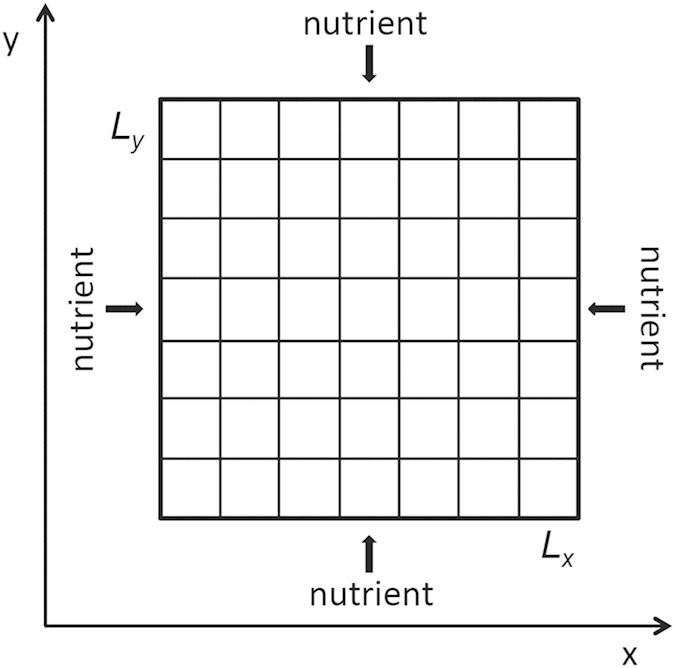
Two-dimensional host tissue discretized by spatial grids. Each grid can contain more than one tumor cell. The nutrients are supplied via diffusion through the borders.

In order to simulate tumor growth, we consider the host tissue represented by a two-dimensional (2D) square lattice of sides Ω = *L*_*x*_ × *L*_*y*_, where *L*_*x*_ = *L*_*y*_ = *L* is the length of each side of the domain (see Fig. 1). The blood vessels around this domain supply the nutrients that are consumed by both healthy and tumor cells^47^,^48^,^49^,^50^. The remainder of this space is discretized to a regular grid in which one or more various cell types reside. Roughly, the grid size is approximately 10–20 *μ*m in size with a dozens actual biological cells.

In our model, the individual cells immerse in the local host microenvironment. For simplicity, we refer to the environmental factors as the extracellular matrix (ECM), which can be degraded by tumor cells. A tumor cell can be placed in the spatial grid only if the density of ECM is *ρ*_*ecm*_ = 0^51^,^52^.

Nutrients are necessary to maintain cell survival and cell mitosis. In fact, the cell also needs many other chemical species in a cell cycle. Here, we reduce the real nutrients and others to a general “nutrient”. Within the simulation domain, the nutrient concentration *ϕ* diffuses and is consumed according to the following reaction-diffusion equation^45^,^47^,^48^,^49^,^50^,


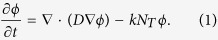


Here, *D* is the diffusion coefficient of nutrients, and *k* represents the rate coefficient of nutrient consumption by one living tumor cell. *N*_*T*_ denotes the onsite population of living tumor cells. The value of *ϕ* ranges from 0 to 1. As mentioned above, the blood vessels around the area of interest supply the stable nutrient source. The conditions at those boundaries for Eq. (1) take on the form


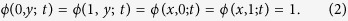


In general, the tumor cells undergo the phenotypic behaviors of proliferation, invasion, quiescence, and death. The genotypes of tumor cells are stable and never change in the lifetime if we neglect further mutational events. However, the phenotypic behaviors of cells are influenced by the cells in the adjacent neighbor site and the local microenvironmental factors^26^,^31^,^36^,^45^,^46^. Cells in death and quiescence do not interact with others. Thus, the remaining phenotypes, proliferation and invasion, have strategic interactions among tumor cells that induce payoff to alter phenotypic behavior^45^.

The payoff matrix of the game between proliferative and invasive cells is defined in Table 1. The left-column denotes a phenotypic cell receiving the reward (the parameter values in Table 1) when encountering another phenotypic cell in the top row. The proliferative probability of a cell will change by *α*_*pp*_ when encountering another proliferative cell or by *α*_*pi*_ when playing against a invasive cell. The invasive probability of a cell will result in changes of either *β*_*ip*_ (against a proliferative cell) or *β*_*ii*_ (against another invasive cell).

Each tumor cell is randomly selected to execute one of the above-mentioned phenotypic behaviors. A minimal local nutrient requirement is necessary to maintain the normal cell behavior, such as division or migration. Thus, a necrotic criterion of tumor cells could be introduced reasonably. If the local nutrient *ϕ* is lower than a threshold *ϕ*_*c*_, living cells in this spatial grid become necrotic or enter a reversible quiescent state. In the other hand, the cell proliferates or migrates whenever the onsite nutrient concentration is *ϕ* > *ϕ*_*c*_^45^. If a cell is marked for division, we propose that the proliferative probability *P*_*p*_ is determined by^45^,^47^,^48^,^49^,^50^


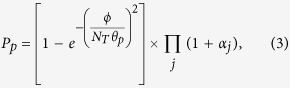


where *θ*_*p*_ is the tunable shape parameter and *α*_*j*_ denotes the interaction between two cells *α*_*pp*_ or *α*_*pi*_ as in Table 1. The first term on the right side of Eq. (3) indicates that the chance of cell division increases on average with the local nutrient concentration per cell, with a sigmoidal curve that is controlled by *θ*_*p*_^47^,^48^,^49^,^50^. The second term represents the influence of the neighboring cells on cell proliferation. In this work, we consider the neighboring cells comprised by the cells not only in the same grid but also in the Moore neighborhood - the eight grids surrounding a central grid in the case of a two-dimensional square lattice.

Similarly, the invasive probability of a selected cell is described by


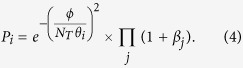


Here, *θ*_*i*_ is the tunable shape parameter and *β*_*j*_ represents the interaction between two cells *β*_*ip*_ or *β*_*ii*_ as in Table 1. The first term on the right side of Eq. (3) indicates that the chance of cell migration decreases with the local nutrient concentration, and the second term comes from the influence of the Moore-neighboring cells. An example of the probability curves of cell proliferation and cell migration is provided in Figure S1 in the supplementary material.

### Algorithm implementation

In the current model, we consider a 2D simulation domain with 401 × 401 (~160,000) grids. Each grid can contain more than one cell. Initially, we place one proliferative tumor cell in the central grid [the *x* − *y* coordinate (201, 201)]. The initial density of ECM *ρ*_*ecm*_ ∈ [0, 1] is assigned in each grid with a uniform probability distribution. The initial density distribution is the same for the initial setup of the nutrient concentration. The whole simulation algorithm is summarized below.

1. *Initialization*. We assign values to all parameters (see the parameter values in Table S1), discretize the studied spatial domain, and initialize the spatial distributions of the ECM and nutrients. Then, a proliferative tumor cell is placed in the central grid, and there are no cells in the remaining tissue.
2. *ECM degradation*. One tumor cell only degrades the ECM density in the located grid with a fixed degradation ability *γ*. The total ECM degradation should take into account the whole contribution of the total living cells in this spatial grid.
3. *Nutrient diffusion*. We solve the PDEs (1) in the studied spatial lattice to obtain the renewed nutrient distributions *ϕ* (see the detailed numerical difference scheme in Text S1).
4. *Determine cell death*. If the updated nutrient concentration *ϕ* in the grid is lower than the critical threshold *ϕ*_*c*_, all of the onsite cells enter the necrotic status. If not, the cell fate remains to be judged in the next step.
5. *Determine cell proliferation or migration*. When *ϕ* > *ϕ*_*c*_ in the current grid, for each tumor cell, we calculate the proliferative and invasive probabilities *P*_*p*_ and *P*_*i*_ with Eqs. (3, 4) and normalize their sum to 1. Here, note that *N*_*T*_ is the living cell number in the local grid and the cell phenotypic attribute involving game interaction is the status at the last iteration round. Now, we provide a new cell phenotypic attribute by uniformly generating a random number in [0,1] to compare with the normalized *P*_*p*_ and *P*_*i*_.
6. *Update cell distribution*. We operate all cells at the same time or update all cell positions synchronously by following the phenotypic behaviors. If the cell phenotype is necrotic, it remains in the current grid forever and is quit of the following simulations. When a cell is selected for proliferation, its offspring will be placed in one of the Moore-neighboring sites or the current site. The daughter cell only stays at the grid with *ρ*_*ecm*_ = 0. If there are several grids with no ECM, the daughter cell prefers to move into the grid with the minimum number of tumor cell (including the necrotic cell) to decrease the cell density. Furthermore, the daughter cell tends to enter the grid with a higher nutrient concentration if there are two or more candidate sites with the same cell amount. In the case of several candidates with the same cell amount and nutrients, the offspring will be placed randomly in one of them. For a cell with an invasive phenotype, its movement direction selection is close to that of cell proliferation. The difference is that the invasive cell only moves into one of the surrounding sites except in the case of *ρ*_*ecm*_ ≠ 0 in the all neighborhood sites (the invasive cell has to stay in the current grid).
7. If the tumor reaches the lattice edge, we stop simulation. Otherwise, we repeat steps (2)–(6) until the terminal running time *t*_*max*_.

### Tumor growth

**Figure 2:**
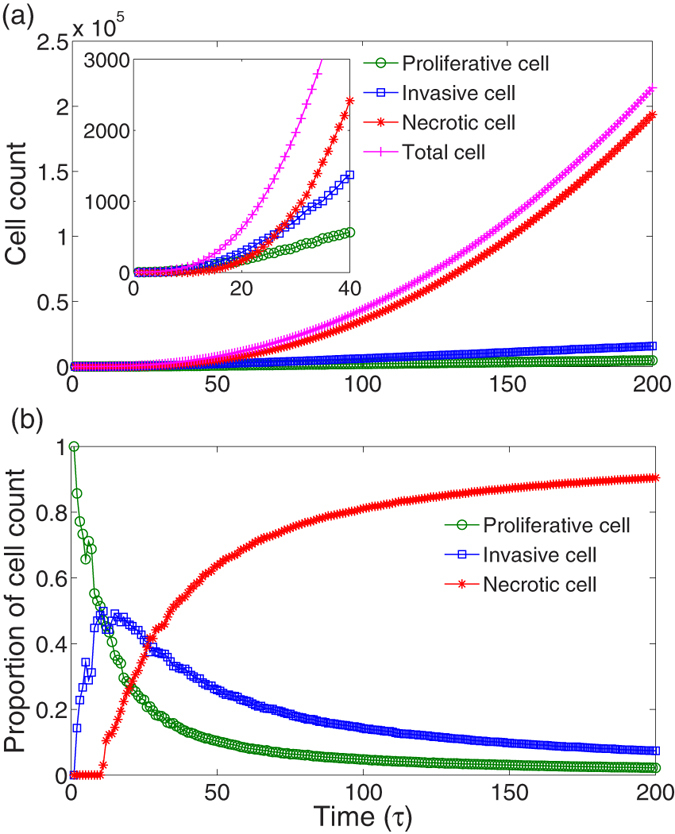
The population of tumor cells over time. (**a**) The total number of tumor cells and the cell counts of different phenotypes increase over time. The inset displays the time evolution of cell numbers in the early stage of tumor growth. With the emergence of necrotic cell, the exponential growth of proliferative and invasive cells become linear at approximately 30 *τ*. (**b**) The time evolution of proportions of different phenotypic cells. Finally, almost all cells are necrotic. Here, the parameters are *α*_*pp*_ = *β*_*ii*_ = 0.1, and the others are the same as those in Table S1.

We perform all simulations from the composite model described above with *t*_*max*_ = 200 *τ* where *τ* is the time interval for updating the cell phenotype once and is similar to a cell cycle. Figure 2(a) shows that the number of tumor cells increases over time. In the beginning stage, the increase of the number of proliferative and invasive cells is exponential but becomes linear at *t* = 30 *τ* because of the emerge of necrotic cells at *t* = 10 *τ*. However, the growth of the total cell count always follows a Gompertz exponential curve. This growth is the same as the evolution of the necrotic cell number. In other words, the exponential growth characteristics of total cell count are decided by living cells but dominated by necrotic cells once cell death appears. These growth properties are consistent with experimental observation and simulation results^49^,^50^,^53^,^54^. This results also means that almost all tumor cells are necrotic. Actually, in Fig. 2(b), the necrotic cells appear after several cell cycles, and the percentage of necrotic cells increases quickly to a stable value of 0.9. In the beginning, the percentage of invasive cells grows faster than that of the necrotic cells. However, it reaches a peak and then quickly decrease to slightly less than 0.1. Moreover, the ratio of proliferative cells to total cells always decreases over time.

**Figure 3:**
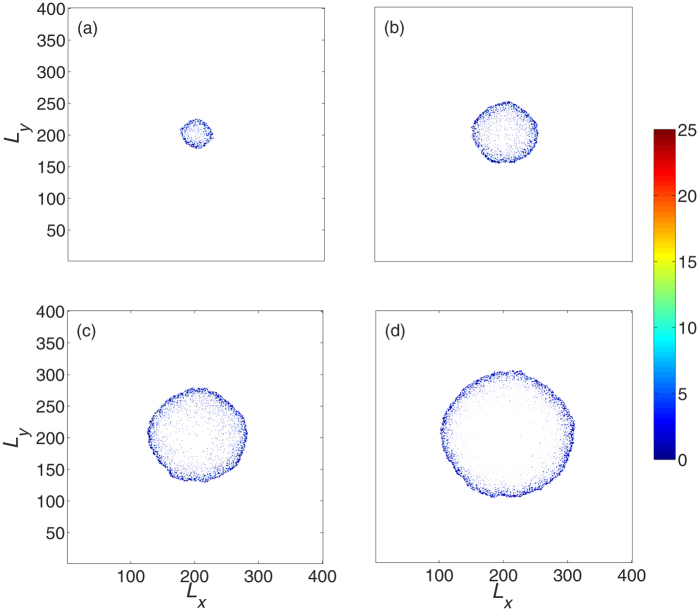
Snapshots of the developed tumor obtained from simulations at different times, (**a**) *t* = 50 *τ*, (**b**) *t* = 100 *τ*, (**c**) *t* = 150 *τ*, (**d**) *t* = 200 *τ*. Note that the necrotic cell and ECM are not on display. The growing tumor exhibits a rough radial symmetry and has an inner necrotic core (see Figure S2). Parameters are the same as those in Fig. 2.

**Figure 4:**
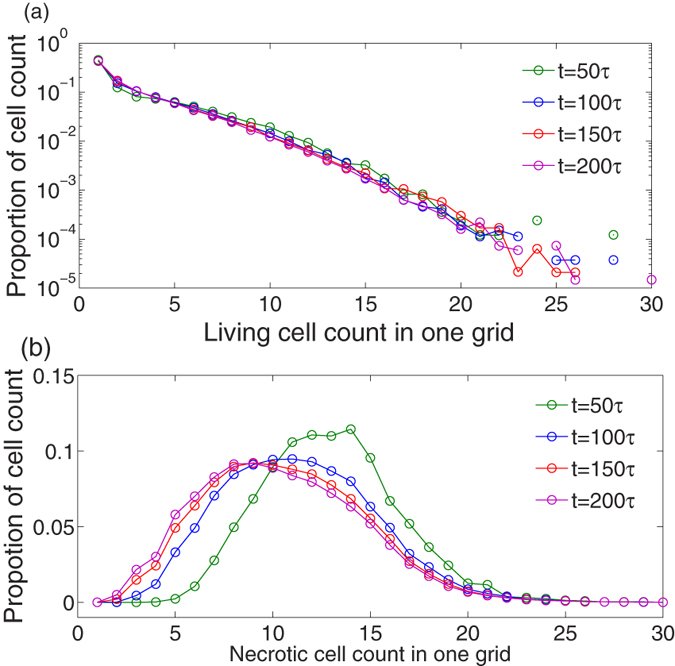
The distributions of tumor cell count in one grid for (a) proliferative/invasive and (b) necrotic phenotypes. The distribution of the living cell count is exponential decay, but the necrotic cell count follows a Gaussian-like form. For living cells, almost all grids contain several individuals (less than 5), but the most hosted necrotic cell counts range from approximately 5 to 16. Parameters are the same as those in Fig. 2.

Figure 3 plots the snapshots of the living tumor cell distribution. Note that we do not display the distributions of the surrounding ECM and the necrotic cells inside the tumor. Clearly, the tumor grows from one cell to a radially symmetric tumor on a coarse scale. The inner part of the tumor is composed of necrotic cells (see Fig. S2) and living cells (proliferative and invasive cells) constitute the outer shell (Fig. 3). The cell density inside the tumor is higher than of the outer shell. As shown in Fig. 4, there is a significant difference between the spatial density of cell numbers for living and necrotic cells. There are few living tumor cells (mostly less than 5) in the grid and the distribution of cell number at each grid decays exponentially [Fig. 4(a)]. However, for necrotic cells, the distribution of cell number is close to a Gaussian curve, and the cell number with maximum probability is ten or more [Fig. 4(b)]. These structural properties mimic the pathological characteristics of avascular tumors^13^,^49^,^53^.

**Figure 5:**
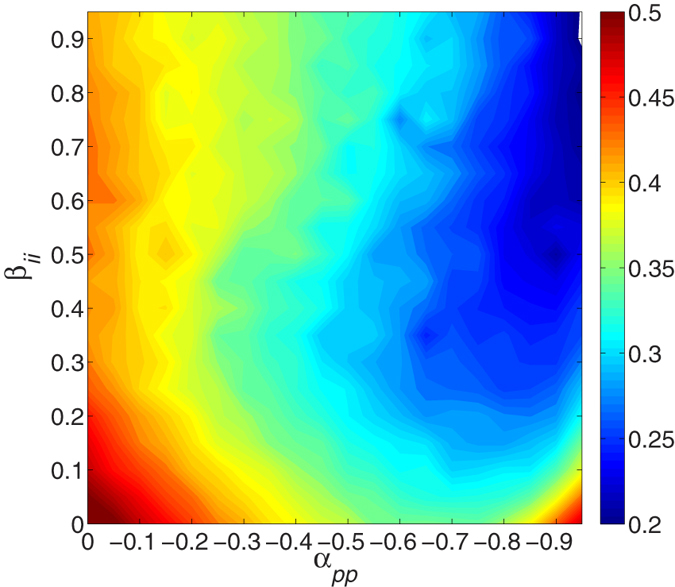
The contour plot of average radial growth velocities 〈*v*〉 over 10 trials with with respect to the interactions between two invasive cells *β*_*ii*_ and between two proliferative cells *α*_*pp*_. If *α*_*pp*_ is fixed in the range of nearly *α*_*pp*_ < 0.9, *v* decreases with increasing *β*_*ii*_ but increases again with further increases, and vice visa for *β*_*ii*_ < 0.3. Other parameters are in Table S1. See text for additional detail.

Considering the rough radial symmetry of tumor growth in the simulation mentioned above, the studied domain can be described by polar coordinates (*r*,*θ*), and the point of origin is the center of the lattice, the Cartesian position of the initially placed proliferative cell. From the radial distances of the tumor front at different times *r*(*t*), one can calculate the growth velocities *v*(*t*) at time *t*. In fact, *r*(*t*) grows linearly over time as observed in the experiments (see Figure S3)^54^,^55^. Thus, *v*(*t*) is almost constant. Figure 5 shows the growth velocities of the tumor front 〈*v*〉 (average over 10 trials) with respect to the enhancement between two invasive cells *β*_*ii*_ and the inhabitation between two proliferative cells *α*_*pp*_. In most cases, there are optimal parameter combinations for smaller *v*. For example, with fixed *β*_*ii*_ in the range of *β*_*ii*_ < 0.3, increasing the inhibition degree *α*_*pp*_ leads to a decrease in *v*, but much more inhibition will induce an increase of *v*. Similarly, this result is also observed in the case of increasing *β*_*ii*_ with fixed *α*_*pp*_ (*α*_*pp*_ < 0.9). Regarding the remaining parameter area, *v* does not display an obvious change with different parameters.

### Surface roughness

**Figure 6:**
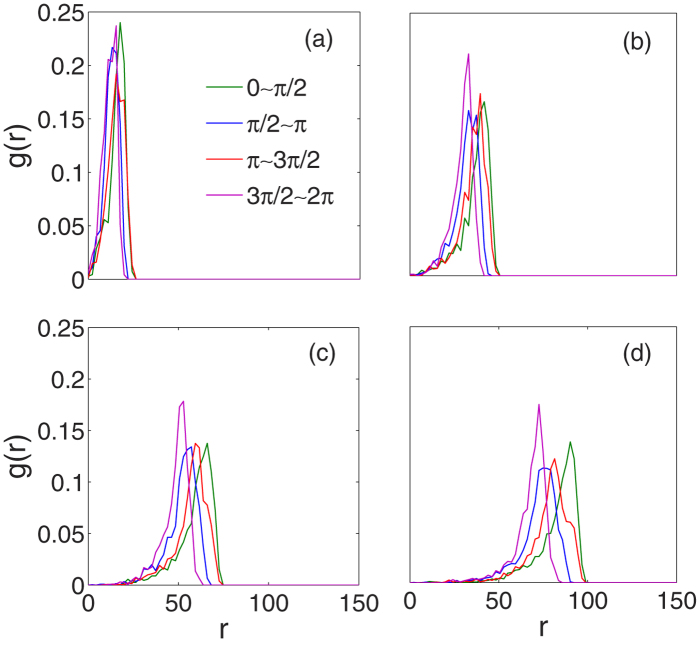
The radial distribution functions *g*(*r*) of cell density along different directions at different times, (**a**) *t* = 50 *τ*, (**b**) *t* = 100 *τ*, (**c**) *t* = 150 *τ*, and (**d**) *t* = 200 *τ*. It is obvious that the shapes of *g*(*r*), both position and peak value, change with directions over time. Parameters are the same as those in Fig. 2.

Tumor morphology is an important criterion in the clinical pathology of cancer. As stated above, the radial symmetry of the growth edge in our simulated tumor is only at a coarse scale. To quantify the anisotropic degree of the growth front, we divide the total domain into four sectors with polar coordinates (*r*, *θ*) mentioned above, *θ* ∈ [0, *π*/2], [*π*/2, *π*], [*π*, 3*π*/2], and [3*π*/2, 2*π*]^51^. The radial distribution function (RDF) *g*(*r*) of the living tumor cells in each sector is defined as^50^,^56^


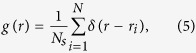


where *N*_*s*_ is the total number of living tumor cells in the calculated sector. Figure 6 shows the RDFs of four sectors changing over time. All the RDFs are asymmetric. Along the radial distance *r*, the interior part of *g*(*r*) increases slowly, quickly peaks and abruptly decreases to zero. This is the fact that the higher density of living cells is at the periphery of the tumor surrounded by ECM or host tissue. Moreover, the peaks of RDFs of four sectors are almost at the same position in the initial stage but widely expand over time. This means the growth velocities and cell density of different directions are different as time goes on. In other words, the tumor surface is no longer smooth.

The surface roughness of the simulated tumors can be studied by tracing the tumor front and transforming the front coordinates from an angle radius into an arc radius. The globe surface roughness at time *t* of the growth front is described as the standard deviation of the radial fluctuations^57^,^58^,^59^,


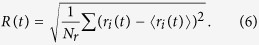


**Figure 7:**
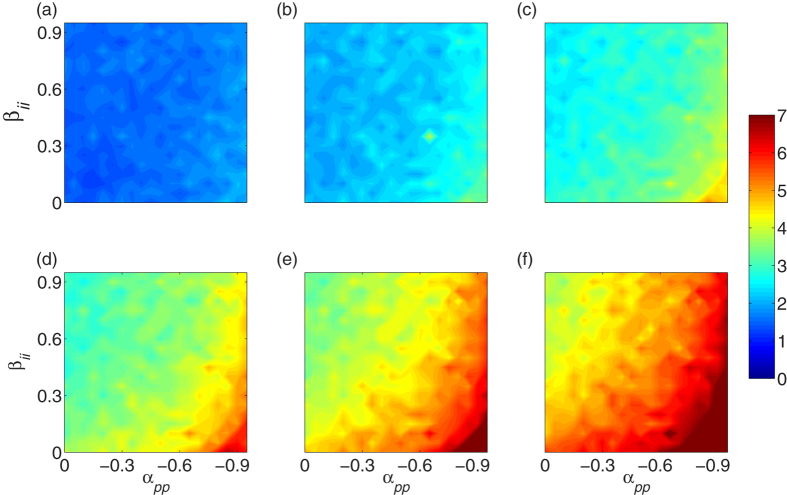
The globe surface roughness *R*(*t*) for various phenotype-phenotype interactions at different times, (**a**) *t* = 30 *τ*, (**b**) *t* = 60 *τ*, (**c**) *t* = 90 *τ*, (**d**) *t* = 120 *τ*, (**e**) *t* = 150 *τ*, and (**f**) *t* = 180 *τ*. *R* always increases over time and is especially stronger with a larger inhibition degree of *α*_*pp*_ and a smaller *β*_*ii*_. Other parameters are the same as those in Table S1.

Here, *r*_*i*_(*t*) is the radial distance of the tumor cell on the surface at time *t*, and *N*_*r*_ denotes the total number of cells occupying the tumor surface. In Fig. 7, we plot the time evolution of *R*(*t*) for various phenotypic interactions. In the early stages of tumor growth, there are no notable differences in *R* and the values of *R* are small [Fig. 7(a)], meaning that in this stage, the tumor has regular symmetry with a smooth surface. However, the values of *R* increase over time in all cases. The higher is the suppressing ability between two proliferative cells *α*_*pp*_ or the lower is the interaction between two invasive *β*_*ii*_, the stronger the roughness *R* becomes over time [Fig. 7(b–d)].

**Figure 8:**
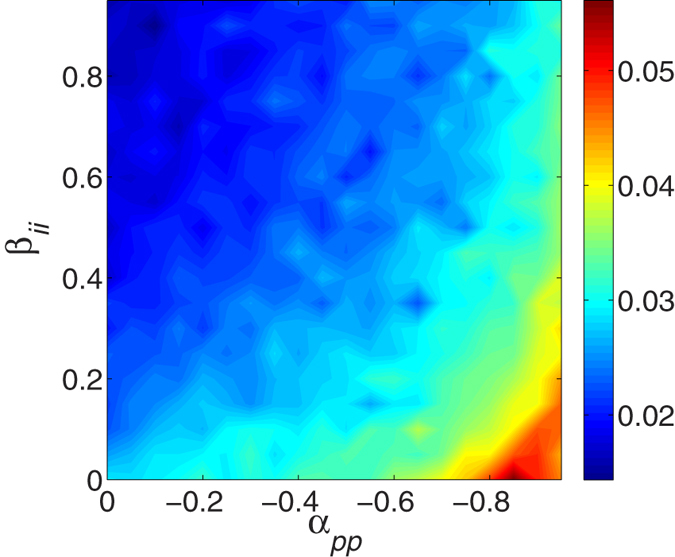
The contour plot of average velocities 〈*v*_*r*_〉 over 10 trials with with respect to the interactions between two invasive cells *β*_*ii*_ and between two proliferative cells *α*_*pp*_. Clearly, larger inhibition between two proliferative cells and smaller enhancement between two invasive cells lead to the slower increase of global surface roughness. Other parameters are in Table S1. See text for additional detail.

Furthermore, we observe that the instantaneous global surface roughness *R*(*t*) always increases linearly with time (see some examples in Figure S4). In other words, the value of *R*(*t*) is found to increase as *R*(*t*) ~ *t* in our simulations. Thus, a constant velocity *v*_*r*_ could be used to quantify the increase of *R*(*t*). Figure 8 provides the average 〈*v*_*r*_〉 over 10 trials for different payoffs of the game among cell phenotypes. It is obvious that 〈*v*_*r*_〉 increases with increasing the inhibition *α*_*pp*_ and decreasing the enhancement *β*_*ii*_.

## Discussion

In this study, we developed an agent-based model to simulate avascular tumor growth, especially involving the game-theory module of phenotypic behaviors. Our coarse-grained model successfully mimics the growth process of avascular tumors with elementary properties. Tumors have a necrotic core and living cells accumulate to form a shell along the growth border.

Our simulation results show that the competition between phenotypic cells, similar to the game behavior between individuals, can influence the growth velocity and surface roughness. The growth velocity decreases with increasing inhibition between two proliferative cells *α*_*pp*_ or with enhancement between two invasive cells *β*_*ii*_. Additionally, the surface roughness of tumors increases over time, and also increases as the *α*_*pp*_ increases (or the *β*_*ii*_ decreases). It shows that the local game interactions distinctly affect the global properties of tumor growth. Actually, even though it is difficult to quantify the interaction between phenotypes because of the complicated genotype-phenotype links, our observations are still helpful for optimally designing experiments or clinical treatment strategies involving different genotypes considering the effect of their phenotypic behaviors.

In our simulations, the linear increase of tumor mean radius is consistent with the 2D *in vitro* experiments^54^,^57^,^58^,^59^. However, the instantaneous global surface roughness *R*(*t*) ~ *t*^*β*^ with *β* = 1 is larger than the *in vitro* experimental result *β* = 0.75 ± 0.05^59^. This difference is partly due to the degradation of ECM in our simulations whereas the cell colony fronts grow in culture medium without other microenvironment in experiment. The another possible cause is the limited simulation time.

Our current model covers most of aspects for tumor growth besides the local phenotypic game among tumor cells, such as nutrient consumption, microenvironmental heterogeneity, etc. Here, we mainly focus on the effect of game behaviors between phenotypes by maintaining sufficient nutrients and the stable degradation of ECM. Our current model is easy to extend to study the effect of the heterogeneous microenvironment by replacing the initial uniform distribution of ECM with other more complex or realistic host structures (For example, Voronoi tessellation)^51^,^52^. Similarly, with slight modifications of nutrient supplies and consumptions, our model can be used to study various tumor phenomena. For example, the nutrient diffusion parameters depend on the direction, or the coefficient of nutrient consumption changes with cell phenotypes and the local nutrient concentration. Furthermore, if it is possible to obtain sufficient and reliable experimental data of different genotypes and genotype-phenotype links, the parameters of phenotypic behaviors in Table 1 can be uniquely determined. Thus, our simulations will produce robust quantitative predictions of tumor growth, which could be potentially valuable for tumor prognosis and individualized therapies.

## Additional Information

**How to cite this article**: Chen, Y. *et al*. Simulation of avascular tumor growth by agent-based game model involving phenotype-phenotype interactions. *Sci. Rep*. **5**, 17992; doi: 10.1038/srep17992 (2015).

## Supplementary Material

Supplementary Information

## Figures and Tables

**Table 1:** The payoff matrix between cells with different phenotypes.

	Proliferative	Invasive
Proliferative	*α*_*pp*_	*α*_*pi*_
Invasive	*β*_*ip*_	*β*_*ii*_
