# Supplementary material for: Simulation of avascular tumor growth by agent-based game model involving phenotype-phenotype interactions
Source: Sci Rep. 2015 Dec 9;5:17992. doi: 10.1038/srep17992 (PMC4673614; doi:10.1038/srep17992)
Supplement: Supplementary Information [file srep17992-s1.pdf]

Simulation of avascular tumor growth by agent-based game model involving phenotype-phenotype interactions: Supplementary

Yong Chen[1,2], Hengtong Wang[3], Jiangang Zhang[4], Ke Chen[5], and Yuming Li[1,+]

[1]Key Laboratory of Digestive System Tumors of Gansu Province, Lanzhou University, Lanzhou 730000, China
[2]Center of Soft Matter Physics and its Applications, Beihang University, Beijing 100191, China
[3]School of Physics and Information Technology, Shaanxi Normal University, Xi'an 710119, China
[4]Institute of Pathology, Lanzhou University, Lanzhou 730000, China
[5]Key Laboratory of Genome Sciences and Information, Beijing Institute of Genomics, Chinese Academy of Sciences, Beijing 100101, China

ABSTRACT

Table S1. Summary of input parameters for our model in simulations.

Parameter	Symbol	Value
Spatial size	L_x, L_y	401
Spatial step	h	0.1
Time period of cell cycle	τ	1
Time step	Δt	0.01τ
Total simulation time	t_{max}	200τ
Diffusion parameter of nutrient	D	1×10^{-4}
Rate coefficient of nutrient consumption per cell	k	0.02
Degradation of ECM per cell	γ	5×10^{-4}
Criteria of nutrient for necrotic cell	ϕ_c	0.02
Shape parameter of proliferative probability	θ_p	0.2
Shape parameter of invasive probability	θ_i	0.2
Inhabitation of proliferation from proliferative cell	α_{pp}	$0 \sim -0.95$*
Inhabitation of proliferation from invasive proliferative cell	α_{pi}	-0.02
Enhancement of migration from proliferative cell	β_{ip}	0.02
Enhancement of migration from invasive cell	β_{ii}	$0 \sim 0.95$*

* with interval 0.05.

Supplementary Figures

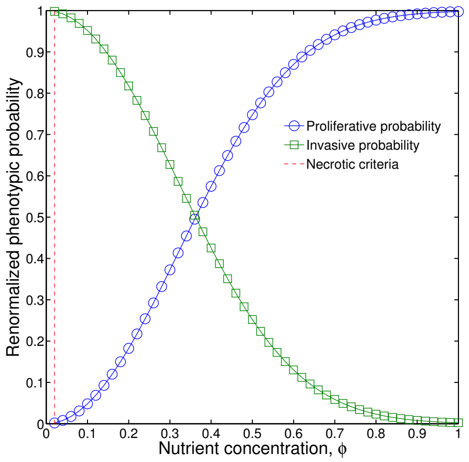

Figure S1. **An example of renormalized proliferative and invasive probabilities for a cell in the spatial grid following by Eqs. (3-4).** Here, the living cell count $N_T = 3$, the shape parameters $\theta_p = \theta_i = 0.2$. The phenotypic parameters in Table 1 are $\alpha_{pp} = -0.1$, $\beta_{ii} = 0.1$, $\alpha_{pi} = -0.02$, and $\beta_{ip} = 0.02$. The blue circled line is the proliferative probability and the green one is of invasive cell. The red dashed line denotes the nutrient criterion of necrotic phenotype $\phi_c = 0.02$.

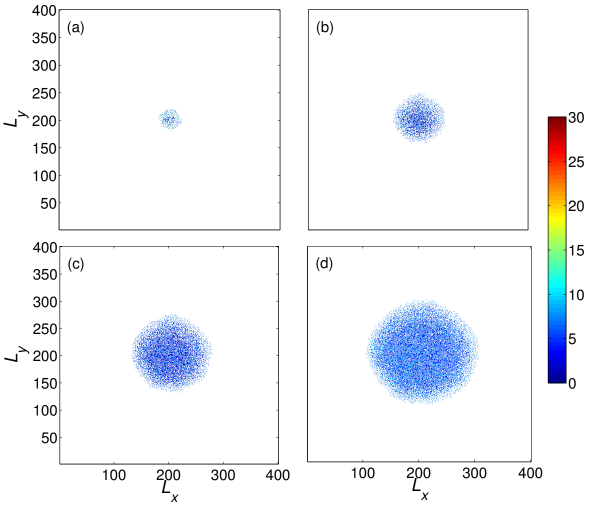

Figure S2. **Snapshots of necrotic cells in growing tumor based on our simulation at different times**, (a) $t = 50\ \tau$, (b) $t = 100\ \tau$, (c) $t = 150\ \tau$, (d) $t = 200\ \tau$. Here, the living cells and ECM have been removed. Parameters are the same as those in Figure 3.

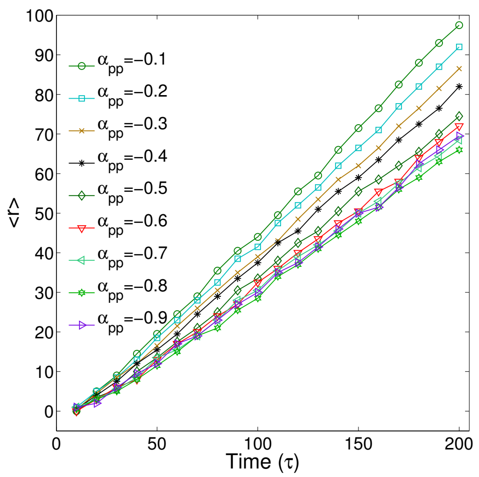

Figure S3. **The average radial distance $\langle r \rangle$ again time** t. $\langle r \rangle$ increases linearly with t. It means the tumor grows with constant radial velocity v. Note that v decreases with increasing the inhibition degree α_{pp} except for $\alpha_{pp} = -0.9$. Here, the parameter $\beta_{ii} = 0.1$ and the others are in Table S1.

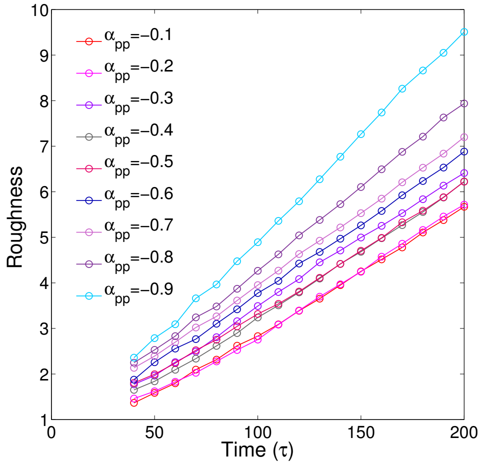

Figure S3. **The average roughness $\langle R \rangle$ again time** t. $\langle R \rangle$ increases linearly with t. It means the surface roughness grows with constant velocity which increases with the inhibition degree α_{pp}. Here, the parameter $\beta_{ii} = 0.1$ and the others are in Table S1.

Text S1. The numerical scheme for Eqs. (1-2).

In this study, the nutrient diffusion equations Eq. (1) is a nonlinear parabolic partial differential equation in two-dimensional space under the conditions Eqs. (2). In the case of one-dimensional space, the similar general parabolic equation is[1]

$$\partial_t u = a\partial_{xx} U + bu, \qquad 0 < x < 1, \quad t > 0. \tag{S.1}$$

The initial and boundary conditions are

$$\begin{aligned} u(x,0) &= g(x), \qquad 0 \le x \le 1 \\ u(0,t) &= \psi(t), \qquad t \ge 0 \\ u(1,t) &= \phi(t), \qquad t \ge 0. \end{aligned}$$

For this class of numerical problem, the differences of Eq. (S.1) are

$$\begin{aligned} \partial_t u\big|_j^n &= \frac{u_j^{n+1} - u_j^n}{\tau} + O(\tau) \\ \partial_{xx} u\big|_j^n &= \frac{1}{h}\left(\partial_x u\Big|_{j+1/2}^n - \partial_x u\Big|_{j-1/2}^n\right) + O(h^2) \\ \partial_x u\Big|_{j-1/2}^n &= \partial_x u\Big|_{j-1/2}^{n+1} - \tau\partial_x\partial_t u\Big|_{j-1/2}^{t_n+\theta\tau}, \qquad 0 \le \theta \le 1. \end{aligned}$$

Here, u_j^n denotes the value of function u at (x_j, t_n). τ and h represent the time and spatial step size, respectively. Substituting the above equations to Eq. (S.1), we have

$$\frac{u_j^{n+1} - u_j^n}{\tau} = \frac{a}{h}\left(\partial_x u\Big|_{j+1/2}^n - \partial_x u\Big|_{j-1/2}^n\right) + bu_j^n + O(\tau + h^2). \tag{S.2}$$

Considering the central differences as below

$$\begin{aligned} \partial_x u\Big|_{j+1/2}^n &= \frac{1}{h}\left(u_{j+1}^n - u_j^n\right) + O(h^2), \\ \partial_x u\Big|_{j-1/2}^n &= \frac{1}{h}\left(u_j^{n+1} - u_{j-1}^{n+1}\right) + O(h^2), \end{aligned}$$

a highly efficient quasi-Saul'ev difference scheme for one-dimensional Eq. (S.1) is deduced,[1–5]

$$\frac{u_j^{n+1} - u_j^n}{\tau} = \frac{a}{h^2}\left(u_{j+1}^n - u_j^n - u_j^{n+1} + u_{j-1}^{n+1}\right) + bu_j^n + O(\tau + h^2). \tag{S.3}$$

A reduced form of two-dimensional parabolic equation used in our simulation could be described by

$$\partial_t u = a\left(\partial_{xx} + \partial_{yy}\right)u + bu. \tag{S.4}$$

With extending the difference scheme of Eq. (S.3), we deduce the numerical scheme,

$$\frac{u_{j,l}^{n+1} - u_{j,l}^n}{\tau} = \frac{a}{h^2}\left(u_{j+1,l}^n - u_{j,l}^n - u_{j,l}^{n+1} + u_{j-1,l}^{n+1}\right) + \frac{a}{h^2}\left(u_{j,l+1}^n - u_{j,l}^n - u_{j,l}^{n+1} + u_{j,l-1}^{n+1}\right) + bu_j^n + O(\tau + h^2). \tag{S.5}$$

After sorting out the above equation, we obtain the final iterative scheme performed in our simulations,

$$u_{j,l}^{n+1} = \frac{1 + b\tau - 2\lambda}{1 + 2\lambda}u_{j,l}^n + \frac{\lambda}{1 + 2\lambda}\left(u_{j+1,l}^n + u_{j,l+1}^n + u_{j-1,l}^{n+1} + u_{j,l-1}^{n+1}\right), \tag{S.6}$$

where $\lambda = a\tau/h^2$.

References

1. Jinpu Lu and Zhi Guan, *Numerical algorithm of partial differential equations* (in Chinese), 2nd edition (Tsinghua press, Beijing, 2003).
2. A. A. Medovikov, High order explicit methods for parabolic equations, *BIT Numerical Mathematics* **38**, 372 (1998).
3. V. K. Saul'ev, On a method of numerical integration of the equation of diffusion, *Dokl. Akad. Nauk SSSR (NS)* **185**, 1077 (1957).
4. V. K. Saul'ev, A new difference method for the numerical solution of fourth-order parabolic equations, *U.S.S.R Comput. Maths. Math. Phys.* **26**, 147 (1986).
5. V. K. Saul'ev, Integration of equations of parabolic type by the method of nets, *International series of monographs in pure and applied mathematics*, Vol. **54**, P ergamon Press (Oxford 1964).
